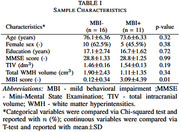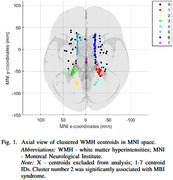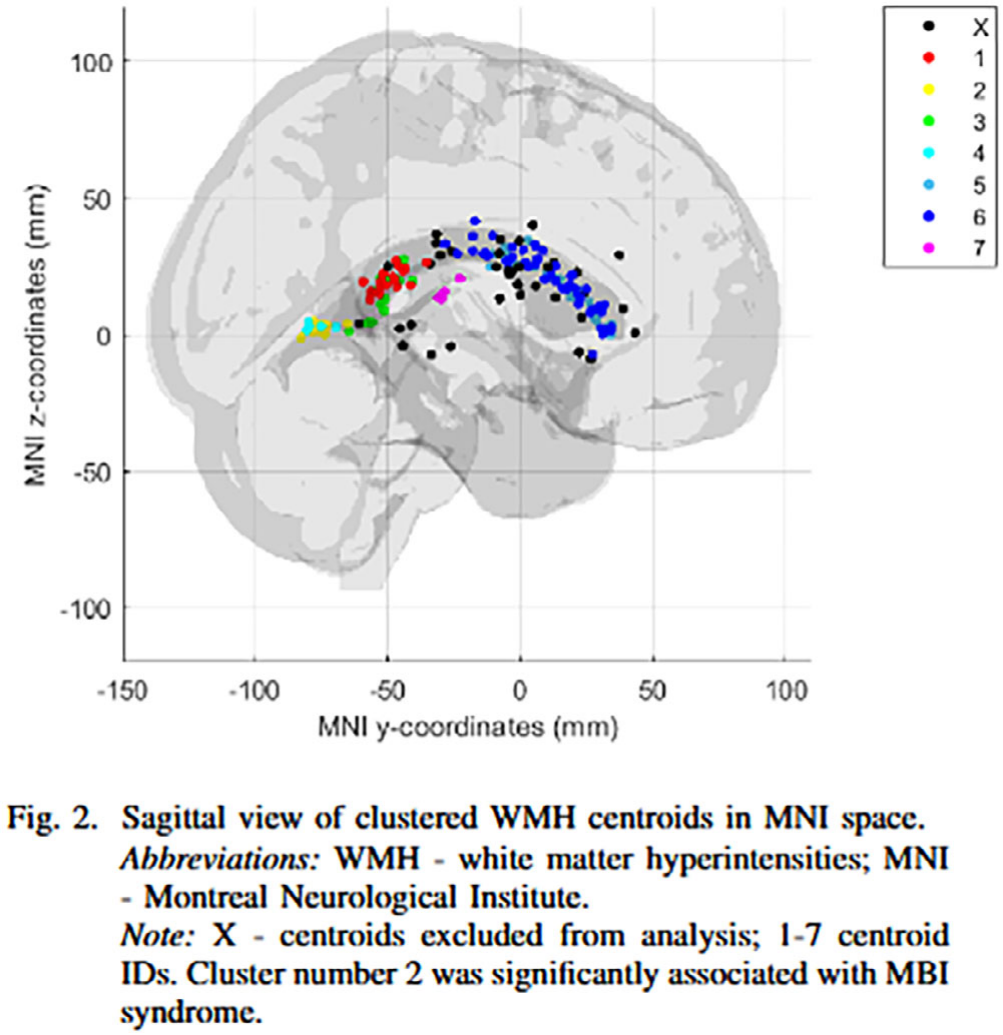# White Matter Hyperintensities and Their Spatial Relationship to Mild Behavioral Impairment: Insights from Cluster‐Based Analysis

**DOI:** 10.1002/alz70856_102637

**Published:** 2025-12-24

**Authors:** Zdenek Linha, Rafael Dolezal, Matej Seifert, Tejasvi Ravi, Pavla Brennan Kearns

**Affiliations:** ^1^ 2nd Medical School, Charles University, Prague, Czech Republic, Czech Republic

## Abstract

**Background:**

Mild behavioral impairment (MBI) is defined by the presence of neuropsychiatric symptoms in cognitively healthy individuals and often predicts the occurrence of dementia. We hypothesize that white matter hyperintensities (WMH), which are a key manifestation of cerebral small vessel disease and are associated with the risk of dementia, may contribute to the development of MBI. This study aims to investigate whether specific WMH lesion locations are associated with MBI.

**Method:**

In our preliminary analysis, a total of 125 WMH lesions from 27 cognitively healthy participants with present WMH lesions (mean age 75 years, 56% females) of the Alzheimer's Disease Neuroimaging Initiative were analyzed (Table 1). The MBI score was derived from Neuropsychiatric Inventory Questionnaire, the MBI syndrome was characterized as having at least one positive symptom of MBI domain persisting for a minimum of six months. WMH lesions from T2‐FLAIR images were automatically segmented with Lesion Segmentation Tool at a 0.95 probability threshold, and their centroids were spatially clustered (Figure 1, 2). Multiple linear mixed‐effects models were employed to predict WMH volumes, lesion spatial distribution or clustered WMH centroids (dependent variables) based on MBI syndrome or MBI scores (independent variables) adjusting for age, sex, education, Mini‐Mental State Examination score, and total intracranial volume.

**Result:**

The analysis revealed a significant association between the MBI syndrome (*p* < 0.001), MBI score (*p* < 0.017), and total WMH lesion volume. Additionally, a negative correlation was observed between the Montreal Neurological Institute (MNI) z‐ and y‐coordinates (*p* < 0.0001), indicating that WMH lesions in MBI‐positive individuals were more likely to localize in posterior superior brain regions. Notably, a cluster including the right lingual gyrus, right calcarine gyrus, corpus callosum, and the inferior occipitofrontal fasciculus demonstrated a significant link with the MBI syndrome (*p* < 0.020).

**Conclusion:**

The association of MBI syndrome with increased WMH lesion volume suggests that cerebral small vessel disease may be involved in the emergence of MBI. The distinct lesion localization in posterior superior brain regions, areas involved in memory recollection, high‐emotion processing, and goal‐oriented behavior, emphasize the importance of spatially resolved WMH analyses in clarifying MBI's neurobiological basis.